# TDP-43 and Limbic-Predominant Age-Related TDP-43 Encephalopathy

**DOI:** 10.3389/fnagi.2019.00376

**Published:** 2020-01-14

**Authors:** Lumi Zhang, Yi Chen, Min Liu, Yunyun Wang, Guoping Peng

**Affiliations:** ^1^Department of Neurology, First Affiliated Hospital, Zhejiang University School of Medicine, Hangzhou, China; ^2^Department of Neurology, Zhejiang University ShuLan International Hospital, Hangzhou, China; ^3^Department of Neurology, Shengzhou People’s Hospital, Shengzhou, China

**Keywords:** TDP-43, neuropathology, Alzheimer’s disease, neurodegeneration, hippocampal sclerosis, dementia

## Abstract

Through a number of an extensive autopsy, biomarker, and genomics studies, researchers have recently defined a novel type of dementia known as limbic-predominant age-related TDP-43 encephalopathy (LATE). LATE is perhaps best characterized by the presence of hyperphosphorylated TDP-43, which plays multi-functional roles through interactions with DNA and RNA, leading to significant alterations in the transcription and translation of particular genes. As individuals of advanced age represent a rapidly growing demographic group globally, there is a steadily increasing rate of LATE incidence that has to date received insufficient recognition despite its serious implications for public health. TDP-43 is the common pathology of various age-related dementia, therefore, it may be a potential and promising therapeutic target for such diseases. In the present review, we discuss the pathways regulating TDP-43 expression, metabolism, and disease activity in order to better understand the link between TDP-43 proteinopathy and LATE at the genetic, pathological, and clinical levels.

## Introduction

TDP-43 is a protein first discovered in 2006 to be present within the ubiquitinated inclusions that are a hallmark of amyotrophic lateral sclerosis (ALS) and in many cases of Tau-negative frontotemporal lobar degeneration (FTLD-TDP; Neumann et al., 2006; Cairns et al., 2007). More recent studies have shown that phosphorylated TDP-43 is similarly present in the brain of individuals over 80 years-old that have not been diagnosed with FTLD or ALS, but who often exhibit sights of Alzheimer’s disease (AD) or hippocampal sclerosis (Amador-Ortiz et al., 2007a,b). These rates of TDP-43 proteinopathy as well as associated hippocampal sclerosis and amnestic dementia have been found to increase at more advanced ages, whereas severe AD cases become less common as individuals attain such advanced age (Nelson et al., 2019). At present, therapeutic efforts have failed to achieve satisfactory outcomes for treating AD, and given its apparent differences from AD, limbic-predominant age-related TDP-43 encephalopathy (LATE) has recently been defined as a unique clinical entity. This new disease classification highlights the importance of thoroughly exploring the role of TDP-43 in the context of age-related dementia development in general, and in the context of LATE specifically. However, as LATE remains a relatively new concept, much of the surrounding literature pertaining to AD or hippocampal sclerosis may inadvertently refer in whole or in part to cases of LATE, thereby complicating interpretations.

## The TDP-43 Protein

TDP-43 is a protein that is 414 amino acids long and 43 kDa in size encoded by the *TARDBP* gene which includes nuclear localization and nuclear export signals, RNA recognition motifs (RRMs) and C-terminal domain (Diaper et al., 2013). The TDP-43 N-terminal domain is important as a regulator of monomer folding and homodimerizing (Zhang et al., 2013). In contrast, the C-terminal domain functions to control gene expression and nucleic acid binding, allowing it to modulate RNA turnover and alternative splicing (Buratti and Baralle, 2010; Lee et al., 2011). Normally, TDP-43 is found primarily in the nucleus of cells, with only 5–20% of TDP-43 being cytoplasmic (Woo et al., 2017), and this imbalance is thought to be autoregulated by negative feedback signaling mediated by nuclear TDP-43 (Polymenidou et al., 2011).

In the context of diseases such as ALS or FTLD, TDP-43 can become cleaved, hyperphosphorylated, and ubiquitinylated such that it aggregates and forms large inclusions within the cytoplasm of cells such as neurons and glia (Neumann et al., 2006). Normally, cellular stress-mediated TDP-43 aggregates are degraded following ubiquitinylation by the caspase-3-mediated proteasome, or through autophagic processes into fragments of 25 or 35 kDa (Chang et al., 2016). However, in pathological contexts this TDP-43 degradation is impaired thereby leading to increased aggregation of 25 and 35-kDa fragments that cannot be eliminated from cells (Huang et al., 2014).

As 25 kDa TDP-43 fragments accumulate, this drives the formation of cytoplasmic aggregates at the expense of normal nuclear localization (Chang et al., 2016). These 25 kDa TDP aggregates have been linked with cognitive deficits (Caccamo et al., 2012), whereas the 35 kDa fragments have been linked to behavioral deficits (Medina et al., 2014). Mutations in TARDBP have been shown to result in inflammation-mediated deregulation of TDP-43 homeostasis which through increased interleukin (IL)-6 levels that in turn drive the formation of aggregates and the progressive deterioration of motor neurons (Swarup et al., 2011; Diaper et al., 2013). TDP-43 expression levels have also been linked to the expression and activity of particular synaptic proteins including synapsin-I and alpha-amino-3-hydroxy-5-methyl-4-isoxazolepropionate (AMPA) receptor subunits (Gulino et al., 2015). As such, when TDP-43 becomes dysregulated, this can result in a cascade of altered signaling events that mediate impaired synaptic transmission, progressive neuronal deterioration, and motor defects (Diaper et al., 2013).

## TDP-43 Related Proteinopathy

Pathological conditions associated with TDP-43 aggregates include: aggregate inclusions within the cytoplasm of neurons and glial cells, pathological swelling or dystrophy of axonal, rounded neuropil grains, diffuse cytoplasmic TDP-43 staining with a lack of normal nuclear TDP-43 staining indicative of pre-inclusions, and in rare cases the presence of TDP-43 inclusions within the nuclei of neurons (Geser et al., 2010). TDP-43-linked neurodegeneration are divided into four categories according to the lesion patterns: perivascular, focal, sub-pial/sub-ependymal, and diffuse in the deep brain parenchyma (Geser et al., 2010).

Various neurodegenerative diseases have been shown to exhibit signs of TDP-43 aggregates, including, dementia with Lewy bodies (Higashi et al., 2007), argyrophilic grain disease (Fujishiro et al., 2009) and corticobasal degeneration (Uryu et al., 2008). Individuals with perivascular TDP-43 pathology also frequently exhibit significant cardiovascular symptoms such as hypertension and cerebral microinfarcts (Geser et al., 2010), with certain chronic vascular disease having the potential to drive the phosphorylation and misfolding of TDP-43. The most prominent and well-understood forms of TDP-43 proteinopathy are FTLD, ALS, and AD.

FTLD is a form of pre-senile dementia that impacts between 0.01 and 0.03% of individuals between 45 and 65 years of age (Bennion Callister and Pickering-Brown, 2014), leading to progressive deterioration of the frontal and anterior temporal brain lobes, finally causing frontotemporal dementia (FTD). The two primary hallmarks of FTLD at the pathological level include hyperphosphorylated tau protein and TDP-43 aggregate inclusions (Neumann et al., 2006).

ALS is a progressive disease best characterized by the gradual and progressive degeneration of motor neuron function, with males and females having respective lifetime ALS rates of 1/350 and 1/500 (Salameh et al., 2015). The presence of altered TDP-43 has been recognized as a hallmark of ALS. As with FTLD, many studies have explored the mechanisms whereby TDP-43 influences ALS pathogenesis, but at present these studies have been inconclusive and have yielded inconsistent results, suggesting more work is needed to establish the therapeutic value of targeting TDP-43 in this disease.

AD is the best-known and most prevalent form of dementia affecting individuals of advanced age. Extracellular amyloid-β (Aβ) plaque deposition, hyperphosphorylated tau aggregates that form within neurons to generate nerve fiber tangles (NFTs) are hallmarks of AD. Recent work has shown that phosphorylated TDP-43 is also evident within the brains of those with AD (Amador-Ortiz et al., 2007a,b).

In certain cases, TDP-43 aggregation and associated pathology may be a secondary consequence of some upstream neurodegenerative, developmental, or stress-induced influence (Nelson et al., 2016). Much as is the case for tau aggregates, however, once these TDP-43 aggregates form, that can significantly and adversely impact normal protein homeostasis and gene expression to increase the risk of a wide range of diseases, thereby facilitating their development and progression (Nelson et al., 2016).

Therefore, TDP-43 proteinopathy is a promiscuous misfoldingopathy and can overlap with each other in many aspects, so it’s necessary to differentiate them for better clinical diagnosis (Nelson et al., 2010). The main differences are risk genes, pathological proteins and types, clinical syndromes, as well as biomarkers. As granulin (GRN), apolipoprotein E (APOE), TMEM106B and many other risk genes present in more than one TDP-43 proteinopathy, it would be a prosperous finding if there are risk genes that specific to a particular subtype. Previous reports found that ALS and FTLD patients had higher TDP-43 levels in cerebrospinal fluid (CSF; Steinacker et al., 2008), despite not so convinced currently, increased TDP-43 in CSF may be related to the involvement of lesions near the ventricle or a sign of disease severity. Other methods like magnetic resonance imaging (MRI) and FDG-PET has been used to aid diagnosis in TDP-43 proteinopathy. As studies indicate that neurodegenerative diseases are related with specific intrinsic functional connectivity networks that varied among individuals, functional MRI studies may help to explain which groups are susceptible to certain diseases based on brain connectivity (Franzmeier et al., 2018).

## The Association Between Late and TDP-43

LATE has been classified as a form of TDP-43 proteinopathy that impacts adults of advanced age regardless of whether or not they exhibit hippocampal sclerosis; LATE neuropathological change (LATE-NC) is characterized by mislocalized and phosphorylated TDP-43 that mainly affects limbic structures (Nelson et al., 2019). According to the anatomical distribution of TDP-43, the simplified staging includes the amygdala (stage 1); hippocampus (stage 2); middle frontal gyrus (stage 3; Nag et al., 2018). An updated staging scheme suggests amygdala (stage 1); entorhinal cortex and subiculum (stage 2); dentate gyrus of the hippocampus and occipitotemporal cortex (stage 3); insular cortex, ventral striatum, basal forebrain and inferior temporal cortex (stage 4); substantia nigra, inferior olive and midbrain tectum (stage 5); basal ganglia and middle frontal cortex (stage 6; Josephs et al., 2016). From a clinical perspective, LATE is very similar to AD with patients exhibiting progressive memory loss (Nelson et al., 2019). One study described AD with different neuropathologic subtypes (typical, limbic-predominant, and hippocampal sparing; Murray et al., 2011), and another study suggested the association between pTau and pTDP-43 is important in the limbic-predominant subtype (Latimer et al., 2019). However, compared with AD, LATE most commonly affects individuals of very advanced age and many cases with “end-stage” AD neuropathologic changes (ADNC) disease actually lack TDP-43 proteinopathy (Nelson et al., 2019). The current definition of LATE-NC includes cases without ADNC and the subtypes of AD brought by Murray et al. (2011) may refer in part to cases of LATE. Despite this, TDP-43 can comorbid amyloid-β and various tau pathologies in the context of LATE-NC. Studies have shown that Aβ deposition can increase TDP-43 phosphorylation and cytoplasmic localization, whereas Aβ clearance prevents TDP-43 propagation (Herman et al., 2011). TDP-43 and Aβ can oligomerize to seed the amyloid oligomerization (Fang et al., 2014). TDP-43 inclusions have also been detected within neurons exhibiting NFTs that are distinct from tau-based NFTs, suggesting that TDP-43 can mediate NFT formation either alone or through tau interactions (Amador-Ortiz et al., 2007b). Further studies are needed to explore the relationships and interactions between TDP-43, Aβ, and tau in order to determine whether these interactions can be targeted to mediate therapeutic treatment of AD and LATE.

LATE-NC shows feature reminiscent of FTLD-TDP Type A often related to HS, TMEM and GRN genetic risk factors, and it’s necessary to discuss the overlap and differences between these two pathologies. As for differences, LATE-NC was more common in the elderly, associated with more marked neuronal and synaptic loss and with greater reactive gliosis, as well as more corpora amylacea and less cortical atrophy (Amador-Ortiz et al., 2007a). Previous studies found the fragments in LATE and FTLD-TDP TypeA were of no difference (Hasegawa et al., 2008). However, a recent study found that different band patterns of the C-terminal fragments can varied among diseases (Hasegawa et al., 2011).

## The Genetic Relationship Between Late and TDP-43

Five risk alleles have been identified to be associated with LATE (Nelson et al., 2019). Both GRN and TMEM106B have been found to be linked to the risk of hippocampal sclerosis and TDP-43 proteinopathy in the context of FTLD (Baker et al., 2006; Boeve et al., 2006; Van Deerlin et al., 2010). Therefore FTLD and LATE may show one common pathologic pathway. The key to this pathway is reduced progranulin levels. Progranulin expression plays a modulatory role in tissue damage within the central nervous system (CNS) to suppress excessive immunity-based microglial activation and protecting neurons from reactive oxygen species and proinflammatory cytokines (Sun and Eriksen, 2011). Both GRN and TMEM106B variations can result in reduced progranulin levels, the former through creating null alleles while the latter function in the presence of GRN mutations (Finch et al., 2011; Murray et al., 2014; Nelson et al., 2015). In addition, there are pieces of evidence that TDP-43 can regulate the stability of the GRN mRNA through interacting with its 3’-untranslated region (UTR), thereby regulating progranulin levels (Fontana et al., 2015). The reduced progranulin level has direct neurotrophic and inflammatory response-modulating functions and higher susceptibility for stress, which is found to play a role in TDP-43 processing and increase the vulnerability of specific CA1 neuron populations (Zhang et al., 2007; Hokkanen et al., 2019). When GRN is depleted, this leads to enhanced caspase-3 activation, which may serve as an initiating event mediating TDP-43 cleavage and associated TDP-43 pathology (Guo et al., 2010).

Beyond these key risk genes, other genes such as TARDBP, valosin-containing protein (VCP), and C9ORF72 have been found to be associated with TDP-43 proteinopathy (Pesiridis et al., 2009; Mackenzie et al., 2011; Wilson et al., 2013). However, what if any relationship these genes may have with LATE remains to be determined.

## The Pathological Relationship Between Late and TDP-43

Previous study has described two types of TDP-43 distribution: limbic and diffuse (Amador-Ortiz et al., 2007b). One research found that a 10-year increase in age was associated with a 1.8-fold increase in the odds of limbic-type relative to being TDP-43 negative (Josephs et al., 2014b). Therefore, limbic group corresponds most closely to the stage I–III (Josephs et al., 2016), i.e., typical LATE. From stages I to III, besides the severity of amygdala TDP-43 immunoreactivity increased across the stages, only the hippocampus and entorhinal cortex volumes progressively declined with increasing stage (Josephs et al., 2014a). Hippocampal sclerosis (HpScl) in LATE cases, is frequently asymmetric, progressing along a rostral-caudal gradient compared with HpScl in AD (Nelson et al., 2019). Perhaps, the interaction of TDP-43 and tau could change and aggravate the HpScl in AD. In stage B2 and B3 of NFTs, hippocampal TDP-43 is associated with more rapid atrophy of this region, while a link between higher NFT stage and more rapid hippocampal atrophy was detected for TDP-43 stages of 0 and 1 (Josephs et al., 2017). However, as many other factors can cause HpScl and above half LATE cases without HpScl, it is hard to definitely tell that TDP-43 accelerate HpScl in LATE, more studies are needed to make clear relationships among TDP-43, HpScl and LATE.

TDP-43 pathological findings can also include the presence of abnormal TDP-43 fibrillary inclusions in astrocytes, potentially suggesting that the blood-brain barrier may be compromised in affected individuals (Lin et al., 2009). Meanwhile, TDP-43 inclusion-sensitive neurons have also been found in the limbic lobe (Amador-Ortiz et al., 2007b).

## The Clinical Association Between Late and TDP-43

Both hippocampal sclerosis and TDP-43-associated pathology are increasingly recognized to adversely impact cognition (Dutra et al., 2015), with these two conditions acting in an additive manner to impair cognitive function (Josephs et al., 2014b). As mentioned before, LATE is associated with progressive memory deficits (Nelson et al., 2019), and recent work suggests that TDP-43 pathology must progress to stage 2 prior to these AD-like dementia symptoms being evident (James et al., 2016).

Increased TDP-43 inclusion levels have been linked to decreased global cognition and faster cognitive decline in a linear manner (Wilson et al., 2013). Combined hippocampal sclerosis and TDP-43 pathology have been linked to impairment of global cognition and episodic and semantic memory, while TDP-43 pathology in the absence of hippocampal sclerosis has been linked only to poorer episodic memory (Lin et al., 2009; Wilson et al., 2013; Nag et al., 2015). Hippocampal sclerosis on its own has been linked to semantic memory deficits (Wilson et al., 2013). One study suggested that subjects with both AD and TDP-43 proteinopathy were more likely to exhibit symptoms of agitation and aggression (Sennik et al., 2017). These results clearly demonstrate that TDP-43 pathology plays a major role in the loss of cognitive function in those of a more advanced age. Moreover, the specific locations of TDP-43 aggregate formation likely impact the associated clinical symptoms. TDP-43 pathology is most frequently evident in the medial temporal lobe, potentially accounting for its clear link with the loss of episodic memory (Wilson et al., 2013). Other studies have also found TDP-43 deposition in the ventral striatum and basal forebrain is linked to poorer performance on memory, language, and executive tests (Josephs et al., 2016).

In all, the symptoms associated with TDP-43-linked pathology depend on the nature of the pathology and the brain regions wherein these aggregates manifest. As such, further studies of the stages and mechanistic basis for TDP-43 progression in the context of pathological and clinical progression are warranted.

## Concluding Remarks and Future Prospects

There is increasing recognition of the prevalence and importance of TDP-43-associated neurodegenerative disease in individuals of advanced age. While many of the studies on this topic to date have demonstrated TDP-43 are associated with various diseases, it is unclear for the most part whether this pathology is causative, promote and/or enhance disease, or instead is a consequence of otherwise related pathophysiology. Although the mechanism is not that clear, Methylene blue and dimebon have been found to inhibit aggregation of TDP-43 in cellular models (Arai et al., 2010) and the former has also been shown to inhibit AD-like Aβ and tau aggregation *in vitro* (Wischik et al., 1996; Taniguchi et al., 2005). Future research should focus on several aspects in order to better understand the TDP-43 and LATE, then to find a potential therapeutic target. There are many ways to be explored, such as the common pathway of the formation of dementia-related protein; the synergy and interaction among Aβ, tau, TDP-43 as well as other related pathological protein; the real role of progranulin in LATE and the mechanism of Methylene blue and dimebon therapy in LATE. In addition, more research is needed to clearly classify the relationship of TDP-43 pathological features with TDP-43 involved regions as well as corresponding clinical syndromes. It is of importance to address the following questions: why pathological change can also present in part of normal individuals? Is it a prediction of people who will develop age-related dementia? As the 35 kDa fragment of TDP-43 caused motor deficits, can LATE cases show motor syndromes in end-stage? No matter TDP-43 is a cause or an effect, what is the upstream or downstream target of TDP-43? Additional elucidation of the role of TDP-43 in ALS and FTLD may shine further light on its role in LATE as well. It is also important that further studies fully explore clinical neuropathological correlations that may guide the diagnosis and treatment of individuals suffering from age-related dementia.

## Author Contributions

GP and LZ conceived and designed the project. LZ and YC wrote the manuscript with inputs from other authors. ML and YW helped to revise the manuscript. All authors reviewed and edited the manuscript and approved the final version of the manuscript.

## Conflict of Interest

The authors declare that the research was conducted in the absence of any commercial or financial relationships that could be construed as a potential conflict of interest.

## Abbreviations

LATE: limbic-predominant age-related TDP-43 encephalopathy
LATE-NC: LATE- neuropathological change
ALS: amyotrophic lateral sclerosis
FTLD-TDP: frontotemporal lobar degeneration with TDP-43 proteinopathy
FTLD: frontotemporal lobar degeneration
FTD: frontotemporal dementia
AD: Alzheimer’s disease
ADNC: AD neuropathologic changes
HpScl: Hippocampal sclerosis
Aβ: amyloid-β
NFTs: Nerve Fiber Tangles
TARDBP: TAR DNA binding protein
GRN: granulin
APOE: apolipoprotein E
TMEM106B: transmembrane protein 106B
ABCC9: ATP-binding cassette sub-family member 9
VCP: valosin-containing protein
RRMs: RNA recognition motifs
AMPA: alpha-amino-3-hydroxy-5-methyl-4-isoxazolepropionate
CSF: cerebrospinal fluid
UTR: untranslated region.
